# Mastering the robot-assisted pyeloplasty learning curve: analysis of operative efficiency, safety, and functional outcomes

**DOI:** 10.1007/s11701-025-02562-4

**Published:** 2025-07-15

**Authors:** Mahmoud Farzat, Florian M. Wagenlehner

**Affiliations:** 1 Department of Urology, Pediatric Urology and Andrology, Justus-Liebig University of Giessen, Giessen, Germany; 2 Department of Urology and Robotic Urology, Diakonie Klinikum Siegen, Siegen, Germany

**Keywords:** Ureteropelvic junction obstruction (UPJO), Robot-assisted, Pyeloplasty, Learning curve

## Abstract

Robot-assisted pyeloplasty (RAPY) for ureteropelvic junction obstruction (UPJO) is a technically challenging procedure. This study evaluates the learning curve by analyzing operative time, complications, and renal function recovery across sequential cases. A retrospective study of sixty consecutive patients who underwent RAPY between 2019 and 2024, performed by a single surgeon in a specialized robotic department, was performed. Cases were divided into three phases: early (first 20), middle (21–40), and late (41–60). Outcomes included operative time, complications (as classified by the Clavien–Dindo system), length of hospital stay, and improvement in glomerular filtration rate GFR. Mean age was 56 years, 57% of UPJO was on the left side. 55% of patients were males, 90% presented with symptoms, and the mean lateral distribution of the affected kidneys in renal scintigraphy statistical analysis was 32%. All preoperative parameters showed no significant differences among the study groups. The mean console time was 91 min. The mean hospital stay was 4.8 days, and the mean bladder catheter days were 4.2 days. Operative time decreased significantly from the early to the late phases (118 ± 28 vs. 65 ± 18 min, p < 0.001). The overall complication rate was 16%, minor complications 11%, and major complications 5%. Major complications (Clavien ≥ III) decreased from 25 to 5% (*p* =0.02) from the early to the late phase. Five patients were readmitted within 90 days after surgery. GFR improvement was consistent across all phases (+ 14.2 mL/min, *p* =0.25). The RAPY learning curve plateaus at 40 cases, with optimized efficiency and safety and significant improvements in both efficiency and safety until then.

## Introduction

Robot-assisted pyeloplasty (RAPY) has become the preferred surgical approach for ureteropelvic junction obstruction in adults (UPJO), offering advantages in precision and postoperative recovery over open and laparoscopic techniques [1]. The growing use of the robotic method in pyeloplasty is attributed to shorter hospital stays, high success rates, and low complication rates [2]. Additionally, the robotic approach contributed to significantly shorter operating times while maintaining excellent results [1, 3]. Success rates reaching 97% were reported [4]. Rates of 85% success were also reported for secondary robotic pyeloplasty [5]. However, the procedure's technical complexity, which requires mastery of robotic suturing, management of anatomic variations, and intraoperative decision-making, presents a significant learning challenge [6]. The parameters used to measure the impact of the learning curve were operating time, complication rates, and success rates [6]. Research has extensively explored the effects of the learning curve in children [7, 8]. Zhou et al. indicated that around 11 cases are needed to reach the mastery stage and roughly 57 cases to attain the proficiency stage [8]. Others suggested mastering 30 cases to become proficient in the procedure [9]. While previous studies have attempted to quantify the learning curve for RAPY, reported case thresholds vary substantially due to methodological heterogeneity and inconsistent outcome measures [7].

The current literature predominantly focuses on the learning curve for pyeloplasty in the pediatric population [10, 11]. This study focuses on the learning curve of pyeloplasty in the adult population, performed by a single surgeon at a referral robotic center. We employ a multidimensional assessment of the learning curve, incorporating operative time, Clavien–Dindo classified complications, hospital stay, and improvement in glomerular filtration rate (GFR). Based on established robotic surgery benchmarks [12], we hypothesize that surgical proficiency, defined by stabilized operative metrics and minimized complications, can be achieved following approximately 40 cases.

## Methods

### Study design and patient selection

We performed a retrospective analysis of all consecutive patients who underwent RAPY by a single surgeon at our referral center between September 2019 and March 2025. The surgeon had no prior experience performing open or laparoscopic pyeloplasty (beyond assisting in a few cases) but had performed over 500 robot-assisted surgeries, predominantly radical prostatectomies. All patients had a clinically relevant upper urinary tract obstruction confirmed by CT/MRI and symptomatic presentation like persistent sporadic flank pain, recurrent infections, kidney pelvic stones, or progressive hydronephrosis. Additionally, patients underwent renal scintigraphy to determine if the obstruction was dynamically relevant and to assess the lateral distribution of renal function. In our department, if the CT scan did not include a urography phase, we performed a contrast media-enhanced pyelography, preferably as a retrograde pyelography, to visualize the upper tract anatomy before surgery. We combined this intervention with the insertion of a DJ catheter so that the DJ catheter will not be inserted blindly during the RAPY surgery. Alternatively, patients underwent an intravenous pyelography. Vascular anatomy (e.g., lower pole arteries) was mapped preoperatively when possible.

### Surgical technique

All cases were performed in the lateral position with the table broken at the patient's hip. Using the Da Vinci X® system, Intuitive Surgical, California, with the 4-arm transperitoneal approach, in line going cranially to caudally, from medial to lateral, almost pararectal with 30° down scope. Additionally, we used a 12-mm assistant trocar next to the umbilicus. We avoided traversing the mesocolon. We reflected the colon to uncover the renal pelvis and identified and preserved the accessory vessels. With the use of indomethacin green (ICG) to better evaluate the blood transfusion of the pyeloureteral junction, we excised stenotic UPJ with cold scissors, spatulated the ureter (2 cm) and pelvis (1.5 cm) for tension-free anastomosis. Pelvic reduction and reconstruction were performed intraoperatively based on renal pelvis morphology but were not systematically documented. In situations where a DJ catheter was positioned before surgery, it remained in place. In other cases, we performed a ureteral stent placement intraoperatively. For patients with a nephrostomy catheter, the nephrostomy was closed right before the skin incision and kept closed until its removal later on discharge, or it was opened in patients with postoperative complications, such as urinoma. No drains were used. The urinary transurethral catheter was removed on postoperative day 4 if the laboratory and sonography workup was uneventful. The DJ stent was removed at 4–6 weeks under local anesthesia.

Patients were divided into three groups according to a chronological threshold to mirror the learning curve: Early (Cases 1–20, 2019–2020), Middle (Cases 21–40, 2021–2022), and Late (Cases 41–60, 2023–2024). Patients' data were collected prospectively in an institutional data bank and were later analyzed retrospectively. Baseline characteristics, including age, gender, BMI, ASA score, preoperative imaging, symptoms, catheter usage, renal scintigraphy results, and GFR, were analyzed. Perioperative and postoperative outcomes, including console time, catheter days, hospital days, complications, and readmissions that occurred within 90 days after surgery, were also recorded and compared among the study groups.

### Statistical analysis

Analyses were conducted using SPSS® v27 (IBM Corp.). For the matched-pairs analysis, both parametric and non-parametric variables were compared using the independent *T* test and the Mann–Whitney *U* test. The ANOVA test was used for normally distributed data; otherwise, the Kruskal–Wallis test was used. All tests were considered statistically significant at *p* < 0.05. The study was approved by the ethics committees of the Westfalen-Lippe Medical Association and received endorsement from the University of Münster Ethics Review Board (2023–500-f-S), conducted according to the Declaration of Helsinki.

## Results

### Patient demographics and preoperative findings

The mean age in our cohort was 56.3 years, and was similar across the study groups (*p* =0.7). The cohort exhibited a balanced gender distribution, with 55% of participants being male. Pyeloureteral stenosis was more common on the left side, without a difference in occurrence among groups (*p* =0.9). 90% of patients presented with symptoms, and only 10% were referred to surgery because of worsened renal function. Clinically, hydronephrosis was observed in 95% of patients based on sonographic findings. Preoperative renal scintigraphy revealed impaired drainage in all cases. The lateral distribution in renal scintigraphy of the affected kidney was 32%, with similar results among the groups (*p* =0.2). Further detailes in Table 1.

**Table 1:** Baseline characteristics and preoperative parameters

	Total (*n* = 60)	Early phase (*n* = 20)	Middle phase (*n* = 20)	Late phase (*n* = 20)	*p* value
Age (years), mean ± SD	56.3 ± 17.5	54.2 ± 18.3	58.1 ± 16.7	56.5 ± 17.9	0.7
Side (left), *n* (%)	34 (56.7%)	11 (55%)	12 (60%)	11 (55%)	0.9
BMI (kg/m^2^), mean ± SD	26.7 ± 4.4	26.4 ± 4.1	27.8 ± 5.3	25.9 ± 3.9	0.3
ASA score ≥ 3, * n* (%)	14 (23.3%)	5 (25%)	6 (30%)	3 (15%)	0.4
Male, * n* (%)	33 (55%)	12 (60%)	10 (50%)	11 (55%)	0.8
Prior DJ/PCN, * n* (%)	20 (33.3%)	8 (40%)	7 (35%)	5 (25%)	0.5
GFR (mL/min), mean ± SD	67.9 ± 12.7	68.5 ± 12.3	65.2 ± 14.1	70.1 ± 11.8	0.4
Symptoms (Pain), * n* (%)	54 (90%)	18 (90%)	17 (85%)	19 (95%)	0.5
Hydronephrosis, * n* (%)	57 (95%)	19 (95%)	18 (90%)	20 (100%)	0.2
CT/MRI, * n* (%)	49 (82%)	15 (75%)	16 (80%)	18 (90%)	0.4
Pyelography^a^, * n* (%)	42 (70%)	12 (60%)	14 (70%)	16 (80%)	0.3
lateral distribution in renal scintigraphy (%), mean ± SD	32% ± 18	38% ± 20	30% ± 16	29% ± 15	0.2

ASA = American Society of Anesthesiologists physical status classification; DJK C instead of K/PCN = Double J stent or Percutaneous Nephrostomy; GFR = glomerular filtration rate; CT/MRI = computed tomography or magnetic resonance imaging

^a^Either retrograde pyelography or intravenous pyelogram (IVP). Continuous variables are presented as mean ± standard deviation (SD). Categorical variables are shown as a number (percentage)

### Operative metrics and learning curve

Early-phase cases in our cohort took nearly twice as long as late-phase procedures (118 ± 28 vs. 65 ± 18 min, *p* <0.001). Hospital stays were the shortest in group 3 (3.2 days) compared to 6.5 days in group 1 and 4.8 days in group 2 (*p* =0.001). Catheter days were also the shortest in group 3 (2.5 days), compared to 6.1 days in group 1 and 4 days in group 2 (*p* =0.001). GFR improvement was evident in all groups, and patients in all groups benefited from surgery (*p* =0.25). Intraoperative Findings, such as stones in the kidney pelvis or ureter duplex, were similar among groups. In 16/60 patients in our study, a lower pole artery was found intraoperatively, with a higher incidence in patients in group 1 (*p* =0.01). Check Table 2 for more details.

**Table 2:** Postoperative outcomes

	Total (*n* = 60)	Early phase (*n* = 20)	Middle phase (*n* = 20)	Late phase (*n* = 20)	*p* value
Operative time (min), mean ± SD	91.7 ± 28.5	118 ± 28	92 ± 21	65 ± 18	< 0.001
Hospital stay (days), mean ± SD	4.8 ± 2.0	6.5 ± 2.1	4.8 ± 1.5	3.2 ± 1.0	< 0.001
GFR improvement (mL/min), mean ± SD	+ 14.2 ± 7.5	+ 12.3 ± 8.1	+ 14.1 ± 7.5	+ 16.2 ± 6.9	0.25
Catheter days, mean ± SD	4.2 ± 2.3	6.1 ± 2.8	4.0 ± 1.5	2.5 ± 1.0	< 0.001
*Intraoperative Findings, n* (%)					
- Stones	5 (8.3%)	3 (15%)	2 (10%)	0 (0%)	0.12
- Lower pole artery	16 (26.7%)	9 (45%)	5 (25%)	2 (10%)	0.01
- Ureterocele/ureter duplex	2 (3.3%)	1 (5%)	1 (5%)	0 (0%)	0.52

Continuous variables are presented as mean ± standard deviation (SD). Categorical variables are shown as a number (percentage)

*GFR* glomerular filtration rate

### Complications and recovery

10% of patients in our cohort experienced a complication, with complications happening in groups 1 and 2 (30% and 20%, respectively) more frequently compared to no events happened in group 3. 2 patients in our study had polyuria after the procedure and one patient had an anastomotic leak which was diagnosed per ultrasound (CDC I). All those events were managed conservatively, with the catheter left in place for a longer period. Overall, 4/60 patients developed pyelonephritis and were treated with parenteral antibiotics (CDC II), of them three were readmitted. One obese male patient under permanent anticoagulation due to cardiac indications developed a subcutaneous hematoma in one of the trocar incisions. The patient had to be readmitted and the hematoma had to be removed in local anesthesia (CDC IIIa). Two patients experienced inadequate urinary drainage despite a properly positioned DJ catheter in the renal pelvis. Laboratory assessments revealed a decline in renal function, and both patients reported flank pain. These symptoms suggested an issue with the DJ catheter inserted during surgery, necessitating its replacement. The procedure was carried out under general anesthesia, resulting in rapid symptomatic improvement and normalization of renal function. Among these two patients, one complication occurred during hospitalization, while the other manifested after discharge. Resulting in five patients (8.3%) being readmitted within 90 days after surgery (three in group 1 and two in group 2; *p* =0.03). Details are given in Table 3.

**Table 3:** Complications and readmissions

	Total (*n* = 60)	Early phase (*n* = 20)	Middle phase (*n* = 20)	Late phase (*n* = 20)	*p* value
Total Complications^a^	10 (16.7%)	6 (30%)	4 (20%)	0 (0%)	0.02
Minor (CDC I-II)	7 (11.7%)	4 (20%)	3 (15%)	0 (0%)	0.08
CDC I (polyuria/leakage)	3 (5%)	2 (10%)	1 (5%)	0 (0%)	0.32
CDC II (UTIs)	4 (6.7%)	2 (10%)	2 (10%)	0 (0%)	0.21
Major (CDC III)	3 (5%)	2 (10%)	1 (5%)	0 (0%)	0.04
CDC IIIa (subcutaneous hematoma)	1 (1.7%)	1 (5%)	0 (0%)	0 (0%)	0.25
CDC IIIb (stent change)	2 (3.3%)	1 (5%)	1 (5%)	0 (0%)	0.52
Readmissions	5 (8.3%)	3 (15%)	2 (10%)	0 (0%)	0.03

^a^Some patients had more than one complication; we listed the most relevant one. CDC: complications according to Clavien–Dindo [24]

## Discussion

Our present study demonstrates that proficiency in RAPY, as evidenced by operative efficiency and patient safety metrics, is attained after approximately 40 cases. This transition was marked by a 45% reduction in mean operative time (from 118 to 65 min), a decrease in major complications (Clavien ≥ III), and significantly shorter hospital stays (from 6.5 to 3.2 days) in the late phase. Notably, these improvements occurred without compromising functional outcomes, as GFR enhancement remained consistent across all study phases.

Our findings align with prior learning curve studies while providing several novel insights [7]. The 40-case proficiency threshold corroborates the lower range of existing estimates, even for more complex types of robotic surgery, such as colorectal surgery [13], suggesting that focused training on specific technical challenges, particularly intracorporeal suturing and crossing vessel management, may optimize the learning process. Zhou et al.'s 57th threshold was tested in a pediatric series, which is difficult to interpret in comparison to our findings due to anatomical and physiological differences between the two populations [8]. Furthermore, the complication profile observed in our series (10%), although primarily minor, demonstrates a more rapid acquisition of safety compared to previous studies, with late-phase major complication rates (0%) similar to those reported in analogous series [11]. This may reflect the standardized adoption of preoperative pyelography and the intraoperative utilization of indocyanine green (ICG) imaging in our protocol [5]. The consistent improvement in GFR across all phases reinforces RAPY's reliability for relieving functional obstruction, supporting its designation as the gold-standard surgical approach [14].

The leakage rates in our cohort were 2 out of 60 patients due to inadequate urinary drainage through the intraoperatively inserted DJ catheter. The insertion of a DJ catheter has been discussed in the literature [15], raising questions about whether it should be performed antegrade or retrograde using an endoscopic device, or inserted pre- or post-surgically [16]. Nevertheless, our results were within the range of others [4], noting that we inserted the DJ intraoperatively in an antegrade fashion, if not positioned before surgery.

While console time across all groups in our series was comparatively shorter than that observed in other reports [17, 18], hospital stay (4.8 days) and catheter days (4.2 days) were relatively longer [19]. The improvement in Glomerular Filtration Rate (GFR) was evident across all groups, and patients within each group experienced benefits from the surgical intervention, consistent with the literature [17]. Major complications in our study, specifically the single subcutaneous hematoma and two DJ catheter changes postoperatively, must be categorized as major, despite their minor impact on the postoperative course of our patients. Our rates are comparable to those of other larger series [17, 20], although this series was conducted in the pediatric population.

Our findings have several practical applications. First, regarding training protocols, the identified learning curve supports structured proficiency-based training curricula, such as those proposed by the European Robotic Urology Section (ERUS)[21, 22], with particular emphasis on cases 1–40. Second, Quality Metrics: Institutions implementing RAPY programs should monitor both efficiency (operative time) and safety (Clavien–Dindo complications) during a surgeon's initial 40 cases. Technical Refinements: preoperative imaging optimization and ICG utilization to better evaluate the vitality and transfusion of the pyeloureteral junction, as employed in this study, warrant consideration as a standard practice [5].

Our study presents robust single-surgeon data but has limitations to consider. The findings may not be fully generalizable due to the high-volume referral center setting. Cost analysis and long-term functional outcomes, like success rates, were not evaluated. Although the surgeon's extensive robotic experience (over 500 cases) likely sped up the learning curve, there was minimal prior exposure to pyeloplasty. Other factors, such as the institutional learning curve and team coordination, which have both evolved since the robotic system's 2019 installation, may have also affected the results. Additionally, pelvic reduction/reconstruction was performed but not systematically documented. There was an uneven distribution of technically challenging cases (e.g., crossing vessels), which was not adjusted for in the analysis. The effect of preoperative stenting (33% of cases) merits further exploration. Various authors have conducted cumulative sum (CUSUM) analyses to assess the impact of the learning curve on laparoscopic or robot-assisted pyeloplasty, focusing solely on operative time [8, 23]. In contrast, we included additional clinically relevant parameters, such as complications, hospital stay, and improvements in glomerular filtration rate (GFR).

## Conclusion

Our findings suggest 40 cases as a meaningful proficiency threshold for RAPY, with significant improvements in both efficiency and safety observed beyond this point. The findings support the implementation of structured training programs and suggest that multidisciplinary outcome assessment provides a superior evaluation of the learning curve compared to operative time alone. Future research should investigate the potential role of simulation in accelerating skill acquisition.

## Data Availability

No datasets were generated or analyzed during the current study.

## Abbreviations

RAPY: Robot-assisted pyeloplasty
BMI: Body mass index
UPJO: Ureteropelvic junction obstruction
CDC: Clavien–Dindo classification
GFR: Glomerular filtration rate
DJ: Double J catheter
PCN: Percutaneous nephrostomy
ICG: Indocyanine green
ASA: American Society of Anesthesiologists
CT: Computed tomography
MRI: Magnetic resonance imaging
UTIs: Urinary tract infection
